# 2520 Proteomics in the early diagnosis of metabolic syndrome in a Hispanic pre-teen cohort

**DOI:** 10.1017/cts.2018.111

**Published:** 2018-11-21

**Authors:** Guillermo T. Viera, Ángel L. Candales, Horacio S. Rivera

**Affiliations:** University of Puerto Rico-Medical Sciences Campus

## Abstract

OBJECTIVES/SPECIFIC AIMS: The objective of the present study is to determine if decreased adiponectin and increased leptin levels are associated with the development of MetS and identifiable endothelial dysfunction in a cohort of Hispanic pre-pubertal children. To do so we propose the following aims: (1) To measure expression of adiponectin and leptin levels in a Hispanic pre-pubertal cohort and determine their correlation with features of the MetS. (2) To perform proteomic analysis in a Hispanic pre-pubertal cohort. (3) Evaluate early onset of endothelial dysfunction and its correlation with expression of adiponectin and leptin levels in a Hispanic pre-pubertal cohort. METHODS/STUDY POPULATION: A cross-sectional pilot study will obtain a random representative sampling of children aged 6–12 years from all geographical areas of Puerto Rico. Children will be assessed regarding pre-pubertal status through Tanner staging and later divided into pre-MetS Versus MetS groups as well as controls. MetS will include children meeting 3 or more of the current International Diabetes Federation (IDF) criteria. Pre-MetS will include children with at least 1 criterion for MetS. Anthropometric data, blood pressure readings, ultrasound-based noninvasive testing for endothelial dysfunction, and laboratory assays will be performed to the study population and data analyzed for correlation. Total adiponectin and leptin levels will be measured using a commercially available quantitative sandwich enzyme-linked immunoassay test. The study will be submitted to the University of Puerto Rico Medical Sciences Campus’ Institutional Review Board (IRB) for approval. Written consent and assent will be obtained from parents and children respectively to ensure patient anonymity. RESULTS/ANTICIPATED RESULTS: We hypothesize that low levels of adiponectin and high levels of leptin will correlate with features of the MetS as defined by the IDF consensus statement, as well as with clinical features of MetS in undiagnosed Hispanic pre-pubertal youth. We also hypothesize that non-invasive testing of endothelial function will correlate both with clinical features of the MetS and with low levels of adinopectin and high levels of leptin. DISCUSSION/SIGNIFICANCE OF IMPACT: The correlation of findings suggestive of endothelial dysfunction and biomarker expression (mainly adiponectin and leptin levels) in a pre-pubertal cohort has yet to be established and could also provide information regarding early atherogenesis in otherwise unidentified youth at risk. Therefore, by using a proteomic approach, this study aims to measure associations between clinical features of the MetS and expression of proteins associated with an adverse cardiometabolic profile in a Hispanic pre-pubertal population. We will concurrently measure the degree of endothelial dysfunction and evaluate whether a correlation exists between previously mentioned protein expression and early onset of dysfunction.

